# Supplementary material for: Investigating the ME/CFS experience through qualitative analysis of memorial entries
Source: PLoS One. 2026 Apr 22;21(4):e0343374. doi: 10.1371/journal.pone.0343374 (PMC13102223; doi:10.1371/journal.pone.0343374)
Supplement: Attachment — Submitted filename: mecfs_qual_response_final.docx [file pone.0343374.s004.docx]

Tanja Grubić Kezele, Ph.D., M.D.

Academic Editor

*PLOS One*

Dear Dr. Grubić Kezele,

We are submitting our revised manuscript “Investigating the ME/CFS experience through qualitative analysis of memorial entries” for reconsideration for publication in *PLOS One.* We have responded to each reviewer comment below. The authors thank the editor and reviewers for the careful review and thoughtful comments.

Reviewer #1: Overall Assessment

This manuscript presents a thoughtful and emotionally resonant qualitative study exploring the lived experiences of individuals with myalgic encephalomyelitis/chronic fatigue syndrome (ME/CFS) as represented through 505 memorial entries on the National CFIDS Foundation website. Using inductive thematic analysis, the authors identify four interconnected domains—systemic neglect and institutional failure, clinical neglect and failures, social disconnection and advocacy, and personal burden and quality of life. The manuscript contributes novel qualitative insights into the psychosocial and systemic dimensions of ME/CFS-related mortality. The paper is generally well organized, conceptually grounded, and written in clear academic prose. The authors appropriately situate their work within prior ME/CFS research and employ a theoretically sound analytic framework (reflexive thematic analysis within an ecological systems perspective). However, the manuscript would benefit from greater methodological transparency, theoretical integration, and refinement of the discussion to enhance its scientific rigor and interpretive depth.

*Thank you for your thoughtful review.*

Major Comments
1. Methodological Rigor and Transparency
Sampling and Data Source:
The use of publicly available memorial entries is innovative but introduces potential selection and interpretive bias. The authors acknowledge this briefly but should elaborate on how such memorials might disproportionately represent more severe cases or those with greater advocacy engagement. A short section on data provenance and limitations of second-hand accounts would strengthen credibility.

*Thank you for this note. We agree that selection and interpretive bias is important in this study and we have expanded on this possibility in our limitations. We have acknowledged that individuals with greater levels of advocacy as well as more severe symptoms may be particularly likely to be listed on the memorial list. In addition, individuals involved with the National CFIDS Foundation, the foundation that manages the memorial list, or other support groups or advocacy organizations may be more likely to be listed as well. We have expanded on these possibilities in our Discussion section:*

*“The analyses relied on existing publicly available data, and secondary data analyses can have several limitations including the original data not being collected for the secondary study and reliance on the data quality of the original source, which may introduce bias or misinterpretation (69). Another important consideration is selection bias, as those placed on the memorial list may have had increased awareness or involvement with the National CFIDS Foundation, or other advocacy or support groups. Similarly, individuals involved in advocacy efforts may have been more likely to be listed on the memorial list, potentially limiting the generalizability of the findings. Individuals with more severe ME/CFS may be more likely to be listed on the memorial list, as some findings have suggested that individuals with more severe symptoms have been suggested to be more involved in self-advocacy (70).” (pages 34, lines 702-711)*

Analytic Process: The manuscript describes inductive coding and theme generation but omits sufficient detail on the codebook development, reflexivity, and researcher positionality. Reflexive thematic analysis (per Braun & Clarke) emphasizes these aspects.

*Thank you for this comment. We have expanded our Methods section to address codebook development and reflexivity. We have also added a research positionality section that describes the background and perspective of each researcher. These sections of the Methods now read:*

*“Coders also identified areas of confusion or lack of clarity in their own coding framework to prepare for codebook finalization in discussion. The coders then met to establish a codebook, discussing the meanings and distinctions of the codes developed. Coders shared their code list and framework, exploring similarities and differences between their coding frameworks. Discussion between coders included identifying foundational codes based on concepts that were expressed repeatedly, as well as subcodes. The coders also explored how and why interpretations of some codes may differ between coders or be unclear, resulting in clarifying and defining these codes. The coders engaged in reflexivity throughout the coding and thematic analysis process, examining their assumptions and potential biases. The coders considered how their educational background, professional training, personal experiences, and underlying assumptions may have influenced their interpretation of the memorial narratives and resulting code development. Discussion of themes between the coders was designed to allow consideration of diverging perspectives, resulting in a more comprehensive understanding of the narratives. Saturation was not assessed as this was a secondary data analysis. Saturation has been criticized as a heterogenous and inconsistently applied concept (28), further supporting its omission from this analysis.*

*Each of the coders independently coded each of the entries before meeting again to discuss emerging initial themes. Each code was dichotomously coded as either “present” or “absent” within each memorial narrative utilizing an Excel document. Examples of these coded memorial entries are shown in Supplementary Table 2, with specific participant memorials removed for privacy purposes. The coders identified associations between codes and resulting themes, as well as associations between themes. Interrater reliability was not assessed, aligning with recommendations for thematic analyses (29). Instead, the coders considered individual interpretations and worked together to develop themes that considered the understanding of both coders. Analytic trustworthiness was promoted through coder discussions in which coders compared their interpretations and reflected on how their background and perspective may influence coding decisions and conclusions. After these themes were identified and named, the researchers read through selections of the entries to ensure that these themes accurately and adequately described the ideas and experiences communicated in the entries. The themes were then finalized with descriptive quotes identified to illustrate the emerging themes. Emerging themes and subthemes are displayed in Table 1, and codes and subcodes are shown in Supplementary Table 1.*

*While all entries analyzed are publicly available (25), full names have been omitted to protect privacy. All quotes are provided verbatim, including original spelling, punctuation, and grammatical errors, to allow for the voice of the individual or their acquaintance to be accurately shared. Although these data were publicly available, memorial narratives are inherently sensitive and personal, and we approached our analyses with care. We chose to remove names and other specific information (i.e., city, insurance company name) to reduce direct identifiability while retaining gendered pronouns (he/she) to allow narrative coherence and respecting the expressive purpose of the original text. (pages 7-10, lines 148-189)*

*“Positionality*

*The authors acknowledge the impact of their identities and experiences in shaping their interpretations and conclusions. The first author is a white, cisgender American woman. She holds a bachelor’s degree in kinesiology and psychology, a Doctor of Physical Therapy degree, a master’s degree in kinesiology, and is pursuing a PhD degree with majors in immunobiology, kinesiology, and psychology. Her academic background informs her focus on the interconnections between physical and mental health in the context of chronic health conditions, seeing all health conditions as not dichotomously physical or mental health issues. She acknowledges her social privilege due to her education and academic training, and she appreciates her lived experience limitations regarding personal experience of underprivileged social identities. She has lived with ME/CFS for approximately six years, contributing lived experience and a patient perspective that may influence how she interprets memorial narratives. The second author is a cisgender American woman with mixed Arab heritage. She holds a bachelor’s degree in psychology, a Master of Social Work degree, and is pursuing a PhD degree in Counseling Psychology. Her academic background and clinical social work experience inform her focus on an ecological systems framework, which emphasizes the impact of external factors on an individual's lived experiences. Her background also informs her focus on highlighting the lived experiences of and addressing the barriers faced by marginalized communities such as those with chronic illness. She acknowledges her social privilege stemming from her academic and social backgrounds, as well as her lack of personal experience in living with chronic illness. She made conscious efforts to check existing biases while interpreting the memorial narratives; however, her held identities may ultimately influence her interpretation of the data.” (pages 10-11, lines 191-211)*

The authors should specify: How disagreements between coders were resolved beyond “discussion.”

*Thank you for this comment. As suggested by Braun & Clarke, we did not assess interrater reliability. However, we engaged in discussion regarding our interpretations as well as codes that we were unsure about. We also reflected on how our experiences and academic background may have influenced our interpretations and conclusions. We have expanded on the development of the codebook and thematic analysis in the Methods section:*

*“A thematic analysis using inductive coding strategies (26,27) was performed to analyze entries on the memorial list. Two researchers, each with experience in coding and interpreting qualitative data, were involved in the coding process. First, the coders familiarized themselves with the data, reading through each of the entries and taking notes regarding potential codes as well as patterns demonstrated in the entries. Coders also identified areas of confusion or lack of clarity in their own coding framework to prepare for codebook finalization in discussion. The coders then met to establish a codebook, discussing the meanings and distinctions of the codes developed. Coders shared their code list and framework, exploring similarities and differences between their coding frameworks. Discussion between coders included identifying foundational codes based on concepts that were expressed repeatedly, as well as subcodes. The coders also explored how and why interpretations of some codes may differ between coders or be unclear, resulting in clarifying and defining these codes. The coders engaged in reflexivity throughout the coding and thematic analysis process, examining their assumptions and potential biases. The coders considered how their educational background, professional training, personal experiences, and underlying assumptions may have influenced their interpretation of the memorial narratives and resulting code development. Discussion of themes between the coders was designed to allow consideration of diverging perspectives, resulting in a more comprehensive understanding of the narratives.” (page 7, lines 144-161)*

Whether NVivo or another tool was used for coding.

*Thank you for this suggestion. We have clarified that each code was dichotomously coded as either “present” or “absent” for each individual memorial narrative within an Excel document. No qualitative methodological or analysis tool was utilized. We also added an additional table to our supplemental material to provide further clarification on our coding process. We have expanded on this approach in the Methods section:*

*“Each code was dichotomously coded as either “present” or “absent” within each memorial narrative utilizing an Excel document. Examples of these coded memorial entries are shown in Supplementary Table 2, with specific participant memorials removed for privacy purposes.” (page 8, lines 165-168)*

How saturation was assessed.

*Thank you for this comment. Saturation was not assessed because this study involved secondary data analysis, and thus we could not engage in primary sampling or data collection, which are central to how saturation has been traditionally evaluated. In addition, recent methodological work has criticized saturation as a heterogeneous and inconsistently applied concept across qualitative methodologies rather than a universal standard (Tight, 2023), indicating that its omission is a valid methodological decision. We have clarified this rationale in the Methods section:*

*“Saturation was not assessed as this was a secondary data analysis. Saturation has been criticized as a heterogenous and inconsistently applied concept (28), further supporting its omission from this analysis.” (page 7, lines 161-163)*

*Tight, M. (2023). Saturation: An overworked and misunderstood concept? Sage Journals, 30(7). https://doi.org/10.1177/10778004231183948*

Any steps taken to enhance analytic trustworthiness (e.g., audit trail, peer debriefing).

*While no formal procedures such as an audit trail were conducted, the research team enhanced analytic trustworthiness through collaborative discussions regarding their individual interpretations and how these interpretations may be biased or influenced by personal background and experiences. The coders compared and reflected on their separate interpretations of the data, considering how their individual perspectives and experiences might influence coding decisions and conclusions. This reflexive process helped to identify potential biases and promote more nuanced and credible analysis. We have now explained this process in our Methods section:*

*“Analytic trustworthiness was promoted through coder discussions in which coders compared their interpretations and reflected on how their background and perspective may influence coding decisions and conclusions.” (page 8, lines 172-174)*

Ethical Considerations:
While the dataset is public, ethical discussion could be expanded. The authors might comment on the potential sensitivity of analyzing memorial narratives and their decision to retain pronouns but omit names.

*Thank you for this important point. We have expanded on our decision to remove names but retain pronouns in our analysis, emphasizing our goal to balance privacy of individual experiences while also respecting narrative coherence and the expressive purpose of the original text. We have also now removed other specific information such as city and insurance company name to further reduce direct identifiability:*

*“While all entries analyzed are publicly available (25), full names have been omitted to protect privacy. All quotes are provided verbatim, including original spelling, punctuation, and grammatical errors, to allow for the voice of the individual or their acquaintance to be accurately shared. Although these data were publicly available, memorial narratives are inherently sensitive and personal, and we approached our analyses with care. We chose to remove names and other specific information (i.e., city, insurance company name) to reduce direct identifiability while retaining gendered pronouns (he/she) to allow narrative coherence and respecting the expressive purpose of the original text.” (pages 10, lines 182-189)*

2. Theoretical Framing
The manuscript references an ecological systems approach (Bronfenbrenner, 1977) but does not operationalize it clearly. Consider explicitly mapping findings to the microsystem, mesosystem, exosystem, and macrosystem levels rather than using the four-level structure as a loose analogy. The analysis could benefit from integrating sociological theories of stigma, chronic illness identity, and medical mistrust to contextualize systemic and clinical neglect (e.g., Goffman’s stigma framework, Kleinman’s explanatory models).

*Thank you for your comments. We have added additional discussion*

*to further operationalize the ecological systems approach and made additions to the*	*discussion to more explicitly connect our findings to the microsystem, mesosystem,*	*exosystem, and macrosystem levels of the ecological framework. We also addressed this*

*comment through adding further discussion of sociological theories including Goffman’s*

*Stigma Framework, Kleinmman’s Explanatory Models, and chronic illness identity to*

*further improve interpretation quality in the discussion. Please see additions to the*

*Introduction and Discussion sections below:*

*“The ecological systems model asserts that an individual's experience is shaped by various levels of impact with the individual at the core. Surrounding the individual is the microsystem: the individual’s immediate environment, the mesosystem: interactions between two or more microsystems, the exosystem: larger social structures, the macrosystem: cultural norms, social policies, and other systemic factors, and the chronosystem: the influence of time on an individual's experience (9,10)”* (page 4, lines 84-89)

*“Similarly, this ecological model has been utilized to enhance health literacy and build understanding of other illnesses such as acute SARS-CoV-2 infection, indicating the importance of understanding the impact of the micro-, meso-, exo-, and macro- systems of the ecological model on an individual’s illness experience (17).”* (page 5, lines 97-100)

*“These findings highlight the significant impact of the macrosystem or larger societal*

*structure and culture on an individuals’ illness experience.” (page 27, lines 545-547)*

*“The prevalence and lasting impact of these negative healthcare experiences, characterizes the significant impact of the ecological model's microsystem and exosystem, or direct and indirect societal systems on the illness experiences of individuals managing ME/CFS. This dissatisfaction with healthcare experience may further be explained by prior research on explanatory models, or the patients’ individual understanding of their illness (36). This research emphasizes the importance of healthcare professional's seeking an understanding of the patient’s individual conceptualization, beliefs, and cultural understanding of their illness, to improve treatment plans, patient-provider communication, and other outcomes (36–38). Participant report in this study highlights a lack of explanatory model usage by clinicians, in which patient input is dismissed or met with disbelief, which may contribute to the poor outcomes and patient-provider relations described above.” (pages 26-27, lines 566-576)*

*“These challenges with dismissal and disbelief from others point to a common experience of those managing chronic illness: stigmatization. The concept of stigma has been broadly researched and can contribute to an altered sense of identity and social ostracism due to negative stereotypes for conditions such as chronic illness, as explored by stigma theory (42). Limited research has been conducted to explore the impact of stigma that lacks visibility, including chronic illness, however, one study proposes the importance of understanding this stigma through a broader socio-cultural framework to highlight the health disparities of those with concealable stigma (43) Further research on stigma within concealable chronic illness found internalized and anticipated stigma from others to significantly and negatively impact an individual’s self-concept, or how they view themselves as someone with chronic illness (44). This emphasizes the multilevel, internal and external, impact of stigma on physical and psychological health outcomes (45). Our findings may further accentuate the need for a broader systems level approach in understanding the effects of social stigmatization in those with ME/CFS.” (pages 27-28, lines 588-601)*

*“This presence and/or lack of social relationships within these narratives provides*

*a representation of the ecological model's microsystem, in which an individual is directly*	*affected by their direct social connections in both positive and negative*

*ways. Additionally, we also see the positive impact of social support, particularly*

*through interactions between microsystems, which represents the mesosystem of the*	*ecological model (i.e. multiple family members engaging in advocacy efforts, or family*	*engagement with healthcare professionals).” (page 29, lines 613-619)*

*“These individual impacts and experiences highlight the individual at the core of their*	*own experience, as referenced by the ecological model with the individual as the*

*foundation with surrounding external influences.” (page 30, lines 639-641)*

*“This delay in diagnosis may complicate an individual's ability to accept and take*

*ownership of a chronic illness identity, which some have suggested*

*is an important step in managing and coping with chronic illness (55).” (page 30, lines*

*650-652)*

*“This emphasizes the immense impact of ME/CFS as an illness, and the layers of social and societal impact that influences the lived experience of each individual.” (page 31, lines 669-671)*

*“Given the complex nature of ME/CFS and the mortality risk of the illness highlighted in this study, it will be important for future research to further explore the experience of this illness through the lens of the ecological model or systems theory more broadly. Further research may expand on specific experiences and systems that contribute to mortality risk, specifically mortality by suicide. This conceptualization may also have clinical implications for those treating patients with ME/CFS, in understanding the interconnected and confounding impact of various systems on the experience, treatment, and outcomes of those with ME/CFS. Overall, our findings suggest that a more nuanced and comprehensive framework, such as an ecological model, is needed to thoroughly and more completely understand the lived experiences as well as address the health disparities and barriers faced by individuals with ME/CFS.” (pages 35-36, lines 743-752)*

3. Interpretation and Discussion

Novelty and Contribution: The study expands upon prior quantitative mortality work (e.g., Jason et al., 2006; Sirotiak & Amro, 2025). However, the discussion often repeats descriptive findings rather than advancing interpretive insights. The authors could discuss how memorial discourse itself functions—both as a site of grief expression and as activism within patient communities.

*Thank you for this suggestion. We further acknowledged our expansion of prior quantitative research through a nuanced qualitative approach. We also incorporated this suggestion by adding a paragraph to the Discussion considering the role of memorial narratives as both grief expression and activism within the ME/CFS community:*
*“The memorial nature of the narratives analyzed is also notable. Memorials have been suggested to provide meaning to acquaintances of the deceased, while also suffering from a lack of control over how emotions and memories that are shared in memorial narratives are framed (65). The potential power of memorial narratives in the context of ME/CFS must be considered as well. Associated with significant stigma and dismissal (66,67), individuals in the ME/CFS community have turned to advocacy, as noted in this qualitative analysis. Others have suggested the role that shared grief and loss can have in shaping movement solidarity and calls for justice (68). Similarly, activism has been suggested to offer a meaning making function for individuals experiencing ambiguous losses (68), which are common in the lived experience of ME/CFS (8,40), and may continue to affect acquaintances even after the individual’s death. Considering these factors, the memorial records analyzed may provide a unique combination of both grief and activism in the context of ME/CFS.” (page 32, lines 687-698)*

*“This work also provides a qualitative insight and nuance to prior quantitative research exploring the mortality risk of ME/CFS (22,23).”* *(pages 34-35, lines 722-724)*

Causality and Attribution: Some memorial narratives attribute deaths to ME/CFS or associated conditions. While these perceptions are meaningful, the paper should more clearly distinguish perceived from documented cause of death to avoid overstating medical conclusions.

*Thank you for this comment. We have adjusted our language throughout the manuscript to emphasize that connections between ME/CFS and associated conditions and death are the reports of the memorial entries made by the deceased’s acquaintances and not medical records or confirmed associations.*

Suicide Contextualization: The rich qualitative material on suicidality could be expanded with references to chronic pain–related suicide frameworks and hopelessness models to connect individual narratives to broader psychosocial theories.

*Thank you for this suggestion. We have expanded our theoretical consideration of suicide mentioned in the memorial entries by considering frameworks developed to better understand suicidality in the context of chronic pain. Theoretical considerations suggested to contribute to suicide risk including impaired feelings of belonging, feeling like a burden, hopelessness, and psychological pain, likely also apply to ME/CFS. Particularly considering the frequent mention of chronic pain in the narratives, considering these frameworks in developing our understanding of suicidality in ME/CFS may be helpful. We have expanded on this consideration in our Discussion section:*

*“The frequent mention of suicide in the narratives is also notable, particularly when considered in the context of similar poorly understood and stigmatized conditions such as chronic pain. Several factors are suggested to contribute to suicidality in the context of chronic pain, including impaired feelings of belonging and feeling like a burden as suggested in the interpersonal theory of suicide in chronic pain (60). Mental defeat has also been suggested as a contributor to suicide in the context of chronic pain (61), which may parallel feelings of hopelessness noted in ME/CFS (40). Indeed, both hopelessness and chronic pain have been independently associated with higher risk of screening positive for suicide risk (62). The “ideation-to-action” framework hypothesizes that pain and hopelessness combine to contribute to suicidal ideation (63). While pain in this model is traditionally understood as psychological in nature, physical and psychological pain are tightly connected (64), suggesting that hopelessness and chronic pain may additively contribute to suicide risk. Particularly considering the frequent mentions of chronic pain in the analyzed memorial narratives, frameworks developed to understand suicidality in the context of chronic pain may also provide useful insight for better understanding suicidality in ME/CFS.”* (pages 32-33, lines 672-686)

4. Presentation and Structure

The Results section is comprehensive but overly lengthy. Condensing exemplar quotations and focusing on the interpretive synthesis rather than repetition would improve readability.

*Thank you for this helpful comment. We have reduced the number of quotations included as well as shortened the quotations when relevant to allow improved readability and interpretive synthesis. Any quotations left were thought to significantly contribute to the understanding of the memorial narratives.*

The Figure 1 conceptual model is valuable; however, it should be simplified visually and accompanied by a concise explanation linking each thematic layer to Bronfenbrenner’s systems.

*Thank you for this comment. We adjusted the figure to increase readability. We also*	*provided a description of the model to improve interpretation and connect our figure to*	*the ecological model.*

*“Fig 1. A structural representation of emerging themes and their interrelationships. This figure is a visual representation of the interconnected layers of the ME/CFS experience. Each layer represents a main theme within our findings, with the individual as well as their personal burdens and quality of life, at the core. Expanding outward is the social disconnection and advocacy theme, which is largely representative of the micro- and meso- systems of Bronfenbrenner’s' Ecological Model. Next, is the clinical neglect and failures theme which aligns with both micro- and exo- systems of the ecological model. Lastly, the outermost layer is representative of systemic neglect and institutional failures which most closely mirrors the macrosystem in the ecological model. Identified subthemes are shown in bolded text.”* (pages 26, lines 518-526)

The Abstract slightly overstates the analytical rigor (“inductively developing a codebook” and “themes emerged within four societal levels”). Consider clarifying that this is a reflexive thematic analysis of public memorial narratives rather than primary participant data.

*Thank you for this comment. We have added to the Abstract to clarify that we performed a secondary data analysis on an existing publicly available dataset rather than our own primary participant data:*

*“This secondary qualitative thematic analysis analyzed 505 entries on the National CFIDS Foundation memorial list, inductively developing a codebook from the publicly available memorial records.” (page 2, lines 36-38)*

Minor Comments
Line 79–82: “underfunded and under-researched” — include citation to NIH funding statistics (e.g., Dimmock et al., 2020) for context.

*Thank you for this suggestion. This citation and another, Mirin et al. (2022), have been added to support this point.*

Line 138: “Interrater reliability was not assessed” — briefly justify this decision per Braun & Clarke (2021) guidance.

*Thank you for this suggestion. We have expanded on this decision in the Methods section:*

*“Interrater reliability was not assessed, aligning with recommendations for thematic analyses (29). Instead, the coders considered individual interpretations and worked together to develop themes that considered the understanding of both coders. Analytic trustworthiness was promoted through coder discussions in which coders compared their interpretations and reflected on how their background and perspective may influence coding decisions and conclusions.” (page 8, lines 169-174)*

Ensure consistent formatting of “CFIDS/ME” versus “ME/CFS.”

*Thank you for this comment. As several terms for the condition are utilized throughout research literature and clinical practice as well as within the memorial list itself (ME/CFS, CFIDS/ME, ME, CFS), we have chosen to utilize the term ME/CFS, following convention followed by the Centers for Disease Control and Prevention. We have clarified this choice in the Methods section:*

*“As ME/CFS, CFIDS, chronic fatigue syndrome (CFS), and myalgic encephalomyelitis (ME) were all treated interchangeably in the memorial list, the condition will be referred to as ME/CFS, aligning with Centers for Disease Control and Prevention convention (1).” (page 6, lines 129-132)*

Typographical and stylistic issues: remove redundant spaces, align reference formatting with PLOS ONE style.

*Thank you for this comment. We have removed redundant spaces and aligned reference formatting with PLOS One style.*

Consider shortening the Acknowledgements section—“The authors have no acknowledgements” is unnecessary; simply omit.

*Thank you for this comment. This comment is now omitted.*

This manuscript offers a valuable qualitative perspective on mortality and lived experience in ME/CFS, addressing an underexplored area of research. However, to meet PLOS ONE’s standards for qualitative rigor and interpretive depth, the authors should strengthen methodological transparency, theoretical grounding, and critical interpretation. With revision, this paper could make a meaningful contribution to the literature on ME/CFS, health inequities, and chronic illness experiences.

*Thank you for your very helpful and thorough review. We have revised the manuscript as suggested by your comments as described in the previous points. We thank you again for suggestions that have significantly improved the quality of our manuscript.*

Reviewer #2: Abstract
Additional symptoms could be included to better capture the breadth of experiences described.

*Thank you for this comment. We have expanded the symptoms of ME/CFS listed in our abstract, now noting pain, sleep issues, and cognitive issues, among the most common additional symptoms noted among individuals with ME/CFS:*

*“Myalgic encephalomyelitis/chronic fatigue syndrome (ME/CFS) is an impairing chronic condition characterized by exhaustion and worsening symptoms following exertion, often accompanied by pain, sleep issues, and cognitive issues.” (page 2, lines 31-33)*

Introduction
This is a well-written section. The authors may wish to consider integrating the following reference to further strengthen the context:
Thornton EJ, Hayes LD, Goodwin DS, Sculthorpe N, Prior Y, Sanal-Hayes NEM. Managing Energy, and Shaping Care: Insights from Adults with Myalgic Encephalomyelitis/Chronic Fatigue Syndrome Through Co-Production Workshops. Am J Med. 2025 Jun;138(6):1001–1009. doi:10.1016/j.amjmed.2025.02.008.

*Thank you for this suggestion. This reference is a valuable resource describing the*

*impact of misunderstanding and stigma in ME/CFS and we have incorporated it into the introduction to strengthen the context of the paper, as suggested:*

*“Research has highlighted several relevant systemic factors that contribute to understanding the experience of ME/CFS, including consistent negative experiences with the healthcare system (12), significant underdiagnosis of the condition (13), stigmatization (14), and lack of specialized care (15).” (page 4, lines 91-45)*

Materials and Methods
This section is well written and requires no revisions.

*Thank you for your review.*

Results
In the opening paragraph, clearly outlining the main themes and subthemes would help guide the reader. The theme titled “Systemic neglect and institutional failure” appears to lack clarity regarding its structure. It is unclear whether this is intended as a main theme, with elements such as “lack of acknowledgement” presented as subthemes. Clarifying this hierarchy would improve readability for all sections.

*Thank you for this comment. For improved readability and to clarify the hierarchy of*

*themes, we have outlined the main themes and subthemes within the opening paragraph*

*of the results section:*

*“Four themes emerged within each of these areas. These primary themes at each level of	experience include personal burdens and quality of life, social disconnection and*

*advocacy, clinical neglect and failures, and systemic neglect and institutional failures,	with each theme having additional subthemes. Personal burdens and quality of life were	further characterized by subthemes of functional impairments, individual illness*

*experiences, and varying degrees of life satisfaction. Social disconnection and advocacy were further clarified by illness impacts on interpersonal relationships, disbelief from others, and illness advocacy. Lack of knowledge and negative health experiences with healthcare providers are subthemes of the larger clinical neglect and failure theme. Lastly, the lack of acknowledgment and lack of investment from larger systems subthemes were characteristic of the systemic neglect and institutional failure's primary theme. Each of these themes also correspond to one or more intersecting levels of the ecological model. The identified levels, themes, and subthemes emerging are described below.”* *(page 11, line 216-228)*

Discussion
The first paragraph should briefly outline the key themes and discuss the main findings, rather than reiterating the introduction or the study’s significance, which should already be clear.

*Thank you for this comment. We have removed the opening paragraph of the discussion*	*to more effectively highlight the key themes and main findings in the discussion, and to*	*limit repetition from our introduction as it already conveys information including prior*	*literature and study significance.*

Line 538 requires additional references.

*Thank you for pointing this out. References have been added for this statement:*

*“Each of these experiences have been supported in the larger ME/CFS literature (32–34).” (page 27, lines 551-553)*

Lines 638–659 also require supporting references.

*Thank you for noting this. We have expanded this section to incorporate references positioning our findings in the context of the larger published literature. This section now reads:*

*“Individuals with ME/CFS experience significant challenges at several environmental levels. From a systemic and institutional perspective, individuals with ME/CFS described on the memorial list face challenges in lack of acknowledgement and funding from insurance and disability authorities, as well as a lack of research funding from federal health agencies. This finding aligns with findings in the broader ME/CFS literature, finding lack of research funding and struggles with disability and insurance providers prominent frustrations among individuals living with ME/CFS (30,31). Barriers are also experienced within the medical system and clinical interactions, with misdiagnosis and misattribution of symptoms common, as well as dismissal and poor treatment recommendations. Medical providers often lack education and experience related to ME/CFS, possibly contributing to poor management of the illness (35). The memorial entries also emphasized misattribution and disbelief within personal relationships, furthering feelings of social isolation. Each of these levels of challenges faced by individuals with ME/CFS can contribute to a complicated illness experience characterized by impairing symptoms, chronicity, financial stress, poor social support, poor quality of life and physical and mental health impairment. Some entries described that the experience of challenges at these various environmental and larger systems levels compound, highlighting the complex nature of living with ME/CFS. Among some individuals, the barriers faced contributed to hopelessness and suicidality, variables also noted in other ME/CFS literature (71).”* (pages 35, lines 725-742)

Additionally, including participant excerpts in the discussion section is not appropriate, instead, these excerpts should be interpreted and discussed.

*Thank you for raising this point. We agree that the Discussion should focus on interpretation rather than the presentation of new data. In this instance, the participant excerpt is not used to introduce additional findings, but to illustrate and clarify an interpretive claim already developed in the Results. Consistent with qualitative reporting conventions, the excerpt functions as an analytic anchor that supports interpretive integration rather than as standalone empirical evidence. We have revised the surrounding text to make this purpose explicit and to ensure the emphasis remains on interpretation. This section now reads:*

*“To illustrate that grief and loss in the context of ME/CFS can be complex and conflicted, we draw on a father’s reflection on his daughter’s death” (page 36, lines 758-759)*

*We note that others have utilized quotations in the Discussion for similar purposes:*

*Bell J, Bailey L, Kennedy D. ‘We do it to keep him alive’: bereaved individuals’ experiences of online suicide memorials and continuing bonds. Mortality (Abingdon) [Internet]. 2015;20(4):375. Available from: https://pmc.ncbi.nlm.nih.gov/articles/PMC4662100/*

Reviewer #3: This study conducted a thematic analysis of the memorial entries from the National CFIDS Foundation memorial list submitted by family, friends, or acquaintances following the deaths of individuals living with CFS/ME. The data for the study are publicly available National CFIDS Foundation memorial list. The manuscript is well-written, well structured, clear in reasoning, premises and conclusions. The writing clearly lays out and justifies the aims of research, the justification, the nature of the methodology applied, and the limitations. The authors have provided detailed descriptions of the themes identified from the memorial entries, and explained their reasoning process, and provided data to back up their conclusions.

*Thank you for your detailed review.*

It might be informative for the readers if the authors included a coding manual, similar to previous thematic analysis studies, (e.g. Picariello, F., Ali, S., Moss-Morris, R., & Chalder, T. (2015). The most popular terms for medically unexplained symptoms: the views of CFS patients. Journal of psychosomatic research, 78(5), 420-426.) to help present methodological steps in a concise and clear way.

*Thank you for this suggestion. We have now included our codebook with subcodes listed as Supplementary Table 1. The themes and subthemes emerging through our analysis, as well as relevant descriptions, are now displayed in the added Table 1. We also included an example of the coded memorial entries to enhance clarity on our coding process, as seen in Supplementary Table 2.*

This revised manuscript represents original work. It is not under concurrent consideration for publication, nor does it contain information that is currently submitted or published elsewhere. The authors are in full control over identifying information. All authors have seen and approved this revised manuscript. No financial interests are reported.

Thank you for your reconsideration of this revised manuscript for publication in *PLOS One.*

Sincerely,

The authors
